# Imitation

**Published:** 1980

**Authors:** Philip Radford

**Affiliations:** General Practitioner Westbury-on-Trym, Avon


### Imitation

**Philip Radford, General Practitioner**
Westbury-on-Trym, Avon

To plagiarise is surely the worst of sins for an author; a person who claims originality after copying the works of others is obviously a deceiver. But to live successfully in the world it is not necessary to be original and sinfulness only enters if originality is claimed falsely. In fact, imitation is one method by which the young develop; a child copies the behaviour of its parents, brothers, sisters and companions. Later, children learn from their school-teachers by copying through educational systems; before the young can be taught to think they must be allowed to imitate. Language and increase in vocabulary comes by hearing others speak. Thus, no blame can be attached to a pre-school child who uses swear words habitually; he has merely been mimicking words used by his elders around him. The young like to ape both the speech and the behaviour of those they meet. So one is hardly surprised at seeing the large numbers of boys and girls who light up cigarettes as they emerge from the local comprehensive school, even after they have been told of the dangers of smoking and have viewed an anti-smoking film.

So mimicry is one component of the growing-up process, with children striving to acquire the communication idioms and activities of the older generation, or at least some of them, with influence coming from the home, school, sports-field and television screen in varying proportions.

Should the imitation urge be exaggerated, however, it can become part of a mental illness, thus further illustrating its sociological importance to man. For instance, a single, copied word or phrase may be repeated again and again, or a movement sequence continued with monotonous and annoying regularity. Or, an elderly and insecure person may roar or bark, lion or dog-like, in an attempt to deter neighbours from interfering in his or her affairs or burglars from entering the house. Suggestions that the neighbours may want to help and that there is no need for them to be frightened off by lions may increase the frequency of animal imitation.

Imitation is by no means confined to human beings; indeed it is essential for the continued survival of some insect species. Thus, bee hawk-moths, which fly by day, resemble bumble bees and so get a measure of protection from bird attack and, similarly, there are hover-flies which look like bees or wasps. Then the common bee-fly gets its name because of its similarity to a bumble bee and there is even a moth, the hornet moth, which is remarkably like a hornet. Mimicry is also found among plants; amongst African succulents, living stones look just like small pebbles in their desert habitat.

Sound imitation undoubtedly plays a part in the lives of many bird species. If incubating blue or great tits or wrynecks are disturbed at their nest-holes they hiss in defiance at the intruder. Snakes are feared by many animals, including man, and they hiss if they are threatened; hence, it may well be that a snake-type hiss will repel the wary, intending predator. Swans and owls also hiss at their enemies and some small mammals, such as cats, will do the same. Again, with people, an audience will hiss at an unpopular politician or the cast of an ill-received first-night play, while a questionable decision by a soccer referee or a cricket umpire in a big match incites a tumult of hissing and booing from the adversely affected supporters. Then there are professional comedians who live by impersonation of others on the stage, some specialising, perhaps, in portraying a particularly well-known personality.

Some bird species are prone to mimicry while singing and, as with man, some individuals are better performers than others. In Britain, starlings are sometimes excellent mimics and individuals may imitate all sorts of locally-heard sounds such as an ambulance siren, a mewing cat or a telephone bell as well as various bird song phrases or calls. I remember a blackcap, one of those delightful woodland warblers, which was a fine imitator, giving notes of blackbird, song thrush, robin and great tit in rapid succession; it would be interesting to know if that imitative form of song was passed on to his descendants. In general, Britain does not have many examples of good bird mimics; there is little of the quality of some African or Australian imitators.

Much more imitative than blackcaps are sedge and reed warblers, especially sedge warblers in this country. On one occasion on a May morning, by a reed-bed, I heard the alternating, harsh alarm calls of a house sparrow and a blue tit yet I could see neither of these birds. To my surprise, I found that it was a cock reed warbler which was making these calls and after a while it relapsed to the usual repetitive, high-pitched chirps and song sequences. Now, is there any advantage to a male blackcap or reed warbler, guarding its breeding territory during May and June, in becoming an efficient imitator? Normally, both birds have characteristic and loud songs, quite sufficient to advertise their presence and attract and hold a mate. Perhaps some bird individuals, like some people, just enjoy their ability to imitate the sounds they hear around them.

Nesting birds holding territories are adults, but young birds are known to imitate as well. If young birds of certain species are reared in isolation in sound-proof aviaries, then the full adult song pattern is not achieved when the male comes into breeding condition. The hearing, and presumably the imitation, of the adult song is necessary, for example, for the full development of the chaffinch's song. Regarding behaviour, young moorhens have been observed to feed and tend the chicks of a later brood of their parents in the same nesting season. As a further instance, as I watched an immature blackbird bathe in a pond one August day, another young blackbird came along and went through just the same motions on dry land.

Doubtless an expert human forger is often a highly intelligent person. It could be that the bird which develops into a good mimic has a greater degree of intelligence than its non-imitative neighbour. Certainly, the song of the bird will have the greater variety and contrast, as compared with the normal, stereotyped phrases. Human beings seem adept at all aspects of copying; maybe proficiency at mimicry has evolutionary advantage after all. It should not be forgotten that Thomas à Kempis' book of spiritual guidance has remained the *Imitation of Christ* through many centuries.

